# Case of Bronchotomy

**Published:** 1819-10

**Authors:** Amasa Trowbridge


FOR THE LONDON MEDICAL AND PHYSICAL JOURNAL.

### *Case of Bronchotomy.* By Dr. AMASA TROWBRIDGE. *Originally read to the Medical Society of New-York.*

MARY ANN DEAN, aged seven years, of the town of Rodman, Jefferson-county, on the 15th November, 1818, at twelve o'clock, M. accidentally drew into the trachea a large dried garden-bean. She was nearly suffocated in the first minutes after the accident. A neighbouring physician was called, who found the child, as he supposed, expiring. In the hurry of proceedings, an emetic of ipecac. was administered; it soon excited the stomach, and threw out a bean. The child at the same time became relieved: there was a belief that the bean was thrown from the windpipe. A messenger had been despatched, to request my immediate attendance. I arrived at nine o'clock, P.M.; found the patient an uncommon intelligent child, possessing much fortitude and consideration. She told me she was much relieved after taking the emetic; but that she then "had a pain in the upper part of the breast, and that it hurt her there extremely to make a long breath; that there was a constant disposition to cough, which she prevented as much as possible, fearing, if the pain came again higher into the throat, it would kill her."

Respiration was disturbed; there was a strong disposition to cough, and rattling in the throat. A bean having been thrown out in puking, I was induced to believe that the symptoms might be occasioned by the irritation the bean had produced, by passing into and out of the trachea, and that it was probably expelled. I left the patient, with directions, if symptoms of the bean's being in the windpipe should return or increase, to inform me; at the same time pointed out the necessity of an operation, to give relief. At six o'clock, P.M. on the 16th, a messenger informed me that the child had suffered several extreme paroxysms of distress, and that the most urgent symptoms of suffocation had prevailed, attended with convulsions. As it was evening, and the patient ten miles from me, and the addi-

---

tional difficulties attending an operation in the night, I delayed visiting till morning.

17th, eight o'clock, A.M. found the child extremely feeble, with quick small pulse, a distress in breathing, and other symptoms, that produced conviction in my mind that the bean was lodged in the bronchia. I immediately prepared for the operation: placed my patient upon a firm table, with a twisted pillow placed under her neck, and the head pressed back; her body and extremities supported and secured by assistants. I made an incision in the direction of the trachea, beginning near the cricoid cartilage, and carried it down two inches, dividing the muscles and integuments. A profuse bleeding was occasioned by a division of the left subclavian vein, which passed obliquely across the trachea: I placed ligatures upon the bleeding extremities. I now began at the third cartilage, and divided the trachea in a perpendicular direction one inch, through three of the rings: the patient respired freely and easily through the opening. The bean not being discovered, I placed a steel distender: this separated the lips of the wound, gave an opportunity for instant inspection of the cavity of the trachea, and the introduction of instruments for extraction of the bean. Some coughing ensued, and a bloody mucus discharged at the wound. My patient was turned upon her side, and permitted to drink milk, which she swallowed with ease. She remained in this situation one hour. Finding but little disposition to cough, I introduced a bent probe, with a scoop point, into the bronchia: this produced coughing, and bleeding from the bronchial vessels. The patient being considerably exhausted, and the bean not making its appearance at the opening, I directed light food to be given occasionally, and delay of searching for the bean, in hopes that it would soon appear at the opening. Left the patient, 3 o'clock P.M. Visited again on the 19th, 7 o'clock A.M.: was informed that the child had taken food, and slept considerably during my absence; that a rattling in the throat and difficulty in breathing had been increasing for the last twelve hours; found her pulse extremely small and quick, cold extremities, laborious respiration, free discharge of mucus at the opening, a feeble action of the left lung, an unusual heaving or rising, and convulsive action, of the right one, a livid countenance, with the usual symptoms that attend a patient in the last stage of pneumonia. I was fully satisfied that the bean was lodged upon the left branch of the trachea; that it impeded the inflation of the left lobe of the lungs; and that the patient would not, from her feeble state, be able to raise it from that position.

I bent a silver wire, twelve inches long, at its centre; formed a loop at the bent end, half an inch in diameter; bent the loop so as to form a hook. This I introduced through the opening, down to the right branch of the trachea, with the hook turned to the right lung: this produced strangulation. After a few efforts made by the child, I suddenly turned the hook to the left side, and drew it up; found the bean was inclosed in the hook; drew it within an inch of the opening, when it slipped from the wire. I withdrew the wire, and introduced a pair of small round forceps, placed them upon the bean, and drew it out. I now withdrew the steel distender, and closed the wound by drawing through the muscles and integuments a ligature, and covered with adhesive plaister. Respiration, and expectoration of bloody mucus at the mouth, easy. A gradual recovery, attended with prickling sensations upon the extremities, with the usual symptoms of resuscitation, appeared for the first twenty-four hours. Directed the inhaling of the vapour of hot water; a cataplasm applied over the wound and neck; mucilages for drink; the extremities to be frequently steamed with warm water; a bleeding in twenty hours, and cathartics, if necessity required it. Left the patient, four o'clock P.M. Visited on the 21st, nine o'clock A.M.: found the patient rapidly recovering; the wound closed externally by the first intention; some pain in swallowing, occasioned by the action of the muscles through which the ligature passed. Since, entirely recovered.

*Remarks.*—In all cases of bronchotomy, the steel distender is preferable to tubes. In placing a tube where there is a substance to be extracted or forced out by coughing, the operator is extremely embarrassed: he has no opportunity to inspect the cavity of the trachea, or to seize the offending substance when it is thrown to the opening. The tube is constantly thrown out, or pushed too deep, into the trachea, or filled with blood and mucus, so that its frequent removal and replacement becomes necessary. The steel distender, with indentations on its outer sides, remains firm where it is placed; opens the incision, so that the cavity can constantly be inspected, the offending substance seen, thrown out by coughing, or searched for by suitable instruments. In the operation, when performed for croup, the distender is preferable to the tube; and the perpendicular incision into the trachea may be made more successful than the transverse one. It is generally admitted, that, in cases of croup, there is, after the third or fourth day of the disease, a membranous lining of the trachea formed, commencing at the larynx, and extending to the bifurcation of the trachea, of considerable tenacity, varying in thickness and density: a quantity of mucus is at the same time constantly emitted from the lungs; suffocation may take place, by the thickening of this morbid membrane, and the lodgment of mucus in its cavity, or for the want of powers in the system to throw it off, or passage in the glottis for this secreted mass of purulent matter, or serous effusion, to be thrown out. No person of experience or sound understanding, would suppose, that a horizontal incision between the rings of the trachea, and placing a tube, could relieve; but, if the incision was made perpendicularly and freely through the rings of the trachea, and a distender placed, if the membrane or morbid mass could not be removed immediately with forceps and scoops, it would give free exit to secreted mucus, and prolong life, till the membrane separated from the trachea, and be easily removed or thrown out. The needle and ligature is preferable to adhesive plaister, for securing a wound in this operation. The want of success in uniting the sides of the trachea, when it is divided perpendicularly, is owing to their not being kept in contact: divided cartilage unites in the same manner, and with as much certainty, as bone, when kept in contact. The act of swallowing disposes the two edges to separate; and, without they are brought firmly together by passing a ligature deep through the adjoining muscles, there is danger that they will remain disunited, and occasion serious difficulties. There is but little inconvenience from blood drawn into the trachea through the incision, when an offending body is lodged in the lower part of the trachea. An offending substance may be drawn into the trachea, lodged near its bifurcation, and remain there for weeks, without producing much distress; but, when raised to the epiglottis, the patient is in a state of suffocation, and may expire suddenly.

